# Initial Coronavirus Disease–2019 Closure Strategies Adopted by a Convenience Sample of US School Districts: Directions for Future Research

**DOI:** 10.1017/dmp.2020.147

**Published:** 2020-05-07

**Authors:** Jeff Schlegelmilch, Claire Douglas

**Affiliations:** National Center for Disaster Preparedness, The Earth Institute, Columbia University, New York; School of International Public Affairs, Columbia University, New York

**Keywords:** COVID-19, infectious disease transmission, mitigation, pandemics, school closure

## Abstract

School closures are an important strategy to mitigate the impacts of a pandemic. But an optimal approach to transitioning from in-person to distance learning approaches is lacking. We analyzed a convenience sample of public K-12 schools in the early weeks of the COVID-19 pandemic in the United States. This initial snapshot provides some insights to inform future research into the variation of strategies across school districts, and would benefit from more rigorous methods to determine true correlations between demographic and geographic factors. Additionally, many of these strategies have evolved in response to ongoing and prolonged public health social distancing measures implemented after this analysis was conducted.

School closures are an important strategy to mitigate the impacts of a pandemic.^1,2^ However, an optimal approach to transitioning from in-person to distance learning approaches is lacking.^3^ We analyzed a convenience sample of public K-12 schools in the early weeks of the coronavirus disease–2019 (COVID-19) pandemic in the United States.

This analysis was conducted as a snapshot of strategies in place between March 16 and March 20, 2020, using a convenience sample of 9 school districts that were closed as part of social distancing strategies (Table 1). The sample was selected based on online availability of district closure and continuity of education plans. Efforts were made to achieve some geographic and socioeconomic diversity in the sample, although this is not exhaustive nor fully representative. Information was obtained from state and school district websites, EdData, the Centers for Disease Control and Prevention, and the National Community Survey, as well as media coverage closures.

**TABLE 1:** School Districts Analyzed

School District	Schools	Students	Median Income (US $)	Student Poverty Rate	Initial Closure Strategy (as of 3/20)
**NYC Public Schools** New York, NY	1800	1 126 501	$57 782	73%	Device-based distance learning, effort to provide devices
**Los Angeles Unified School District** Los Angeles, CA	1009	621 414	$68 272	80%	Device- and free resource–based distance learning, effort to provide devices and broadband access
**Montgomery County Public Schools** Montgomery County, MD	212	165 267	$103 178	34%	Device-based distance learning, utilities to provide free or subsidized broadband
**Baltimore City Public Schools** Baltimore, MD	161	79 187	$46 641	50%	Packet-based learning, limited access to devices and broadband
**Seattle Public Schools** Seattle, WA	104	53 627	$79 565	29%	Packet- and free resource–based learning, limited access to devices and broadband
**Pasco County Schools** Pasco County, FL	96	75 001	$48 289	56%	Device-based distance learning, effort to provide devices and broadband
**Port Angeles School District** Port Angeles, WA	8	3701	$48 002	--	Extended spring break, no instructional support
**Hastings Area School District** Barry County, MI	6	2571	$57 312	47%	Packet-based learning, limited access to devices and broadband
**Belleville Township High School District 201** Belville, IL	3	4978	$60 301	45%	Device-based distance learning, effort to provide devices and broadband

The implementation of distance learning was extremely varied, although our initial analysis suggests some insights to explore further. Whether or not these constitute broader patterns warrants further research.

One approach treated the closures as a prolonged spring break, sending students home without supplemental education material or clear plans for transitioning to distance learning. This approach was observed in a small low-density, rural school district. This district had lower reported instances of COVID-19 at the time of implementation. Other school districts provided students with study packets, accessed either online or distributed/picked up at school. This approach was seen in low- to middle-income communities and less affluent suburban counties. Several school districts cited students’ lack of access to reliable Internet and devices as a reason to use study packets instead of online instruction.

More affluent urban and suburban schools used online instruction platforms. Some used a blend of worksheets, online resources, and online instruction, while schools attempted to provide online-capable devices to students in need. Some broadcasted educational material through public access TV channels, social media, and on their websites, while arranging device distribution. Some were also working with Internet service providers to provide low- or no-cost Internet access. One district employed Wi-Fi-equipped school buses throughout the community for students to access.

The 1 constant across school districts was to continue to provide meals for students using free or reduced meal programs. The predominant strategy had students or parents picking up bagged food to take home, although some districts delivered bagged meals via regular school bus routes.

This initial snapshot provides some insights to inform future research into the variation of strategies across school districts and would benefit from more rigorous methods to determine true correlations between demographic and geographic factors. Additionally, many of these strategies have evolved in response to ongoing and prolonged public health social distancing measures implemented after this analysis was conducted. Further evaluation of these strategies, their adaptations over time, and their impacts on students over a wider sample could provide important insights into strengthening educational distance learning strategies as part of broader public health disease control strategies in a pandemic.
